# Local strain effects on bandgap energy in flexible *h*-WO_3_ nanowires

**DOI:** 10.1093/jmicro/dfaf050

**Published:** 2025-10-31

**Authors:** Sho Nekita, Naomu Sekiguchi, Yuya Kasamura, Itsuki Misono, Yusuke Shimada, Satoshi Iikubo, Tetsuya Okuyama, Satoshi Hata

**Affiliations:** Interdisciplinary Graduate School of Engineering Sciences, Kyushu University, 6-1 Kasugakoen, Kasuga, Fukuoka 816-8580, Japan; Interdisciplinary Graduate School of Engineering Sciences, Kyushu University, 6-1 Kasugakoen, Kasuga, Fukuoka 816-8580, Japan; Interdisciplinary Graduate School of Engineering Sciences, Kyushu University, 6-1 Kasugakoen, Kasuga, Fukuoka 816-8580, Japan; Panasonic Industry Co., Ltd., 1006, Oaza Kadoma, Kadoma, Osaka 571-8506, Japan; Interdisciplinary Graduate School of Engineering Sciences, Kyushu University, 6-1 Kasugakoen, Kasuga, Fukuoka 816-8580, Japan; Faculty of Engineering Sciences, Kyushu University, 6-1 Kasugakoen, Kasuga, Fukuoka 816-8580, Japan; Faculty of Engineering Sciences, Kyushu University, 6-1 Kasugakoen, Kasuga, Fukuoka 816-8580, Japan; Faculty of Engineering Sciences, Kyushu University, 6-1 Kasugakoen, Kasuga, Fukuoka 816-8580, Japan; Faculty of Engineering Sciences, Kyushu University, 6-1 Kasugakoen, Kasuga, Fukuoka 816-8580, Japan; The Ultramicroscopy Research Center, Kyushu University, 744 Motooka, Nishi, Fukuoka 819-0395, Japan

**Keywords:** electron energy loss spectroscopy, 4-dimensional scanning transmission electron microscopy, bandgap energy, tungsten oxide, nanowires, density functional theory

## Abstract

According to theoretical predictions, local strain in the bent regions of flexible nanowires can alter their electronic structure. However, the experimental validation of such strain-induced effects remains elusive. In this study, we established a clear correlation between local structural deformation and electronic properties in bent hexagonal-WO_3_ nanowires using 4-dimensional scanning transmission electron microscopy and electron energy loss spectroscopy. Although a simple geometric bending model predicts an expansion of the (0001) lattice spacing on the outer side of the bend, our direct observations revealed a larger expansion than predicted. This lattice expansion was accompanied by a significant reduction in bandgap energy. We employed density functional theory calculations and crystal orbital Hamilton population analyses to provide a theoretical framework for these findings. These results provide direct experimental evidence of strain-induced modulation of the electronic structure in metal oxide nanowires.

## Teaser

We demonstrate that bending-induced strain in hexagonal WO₃ nanowires modulates their bandgap through lattice expansion on the tensile side. 4D-STEM, EELS, and DFT+U analyses jointly uncover a direct link between local geometry and electronic structure, highlighting strain as a powerful tool for electronic tunability in oxide nanostructures. Figure 5.

## Introduction

Metal oxide (MO_*x*_) semiconductors are typically brittle and susceptible to mechanical failure because of their inherently low mechanical toughness in bulk form. They exhibit significantly enhanced mechanical flexibility when engineered into low-dimensional structures, such as nanowires or nanosheets [1, 2]. This improvement has attracted considerable attention for applications in flexible electronic devices, such as photodetectors [3], artificial skin [4] and strain sensors [5], where high mechanical flexibility is required alongside diverse functional properties. The optical, electrical and chemical properties of electronic devices depend on the electronic states of the semiconductors they incorporate, such as bandgap energy (*E*_g_) and impurity levels [6, 7]. Cathodoluminescence (CL) is a spectral method used in conjunction with scanning electron microscopy (SEM) to investigate the electronic states of semiconductors [8–10]. This method generates carriers with an electron beam and observes their relaxation in semiconductors. This process enables the evaluation of *E*_g_ in direct-bandgap semiconductors [9, 11], and the detection of luminescent defect states with localized energy levels [10]. However, CL’s application tends to be limited in indirect bandgap semiconductors or nonradiative defect states because of their low emission efficiency.

To address this limitation, electron energy loss spectroscopy (EELS) combined with scanning transmission electron microscopy (STEM) provides a powerful alternative. STEM–EELS enables the analysis of electronic states with high-spatial-resolution through excitation events such as interband transitions [12, 13], bulk plasmon excitations [14] and exciton generation [15], regardless of luminescence efficiency. With recent advances in monochromator technology, STEM–EELS can now achieve energy resolutions as low as 20 meV, and in the most advanced instruments, even below 5 meV [16, 17], making it possible to detect subtle energy shifts in excitation phenomena. Monochromated STEM–EELS has been used to experimentally detect variations in the excitation energy of bulk plasmons in bent regions of GaAs nanowires [18] and changes in the electronic structure of heterostructured nanowires [19].

The electronic properties of semiconductors are intricately influenced by their local atomic arrangements and lattice constants [20–26]. Therefore, understanding the electronic properties of semiconductors requires a thorough analysis of their local microstructural characteristics. Four-dimensional STEM (4D-STEM) is an effective technique for evaluating such microstructures at the nanoscale level. This method uses an electron probe to obtain annular dark-field (ADF) images while simultaneously collecting a series of diffraction patterns via a camera. Compared with conventional high-resolution (S)TEM imaging, 4D-STEM offers structural information with significantly lower electron beam exposure, making it particularly suitable for beam-sensitive materials [27, 28].

Several theoretical studies have suggested that the electronic structure of nanowires can be locally modulated by microstructural variations in bent regions [29]. Since strain and lattice distortions can modulate the band structure of MO_*x*_ materials, it is essential to conduct both local structural analysis and spatially resolved measurements of electronic properties to elucidate the underlying mechanisms. Although the effects of strain on band structure have been extensively studied using optical techniques [30, 31], such approaches cannot provide direct evaluation of the structural versus electronic properties of nanostructures like nanowires, where the diameter is less than a few hundred nanometers. In this study, we aimed to clarify the correlation between local structural deformation and the electronic properties in bent hexagonal tungsten oxide (*h*-WO_3_) nanowires, which are representative MO_*x*_ materials with promising applications. The *h*-WO_3_ nanowire offers several advantages for next-generation gas sensors and optoelectronic devices. These advantages include a tunable high-aspect-ratio morphology [32], excellent thermal and chemical stability, superior mechanical flexibility, high performance gas sensors [32, 33], electrochromic behavior [34] and strong catalytic activity [35].

To elucidate how local mechanical deformation influences the electronic properties of *h*-WO_3_ nanowires, we first performed 4D-STEM analysis to measure the nanoscale strain induced by bending. This technique allowed for a critical comparison between the experimentally measured strain and the values predicted by a simple geometric bending model. Second, we utilized first-principles calculations combined with crystal orbital Hamilton population (COHP) analysis to predict how local structural variations modulate the band structure. These theoretical predictions were directly validated at the nanoscale by performing high-spatial-resolution *E*_g_ measurements using monochromated STEM–EELS.

## Experimental

### Hydrothermal synthesis and sample preparation

*h*-WO_3_ nanowires were synthesized using a previously reported method [32]. All chemicals were used as received without further purification. Tungsten powder (Sigma-Aldrich, 99.9%) was first dissolved in hydrogen peroxide solution (FUJIFILM Wako Pure Chemical Corp., 30–35.5%). Excess hydrogen peroxide was then decomposed by immersing a platinum foil (SANYU Electron, 99.99% purity) in the solution. After diluting the solution with deionized water to achieve a tungsten ion concentration of 28 mM, oxalic acid (Sigma-Aldrich, 99.0%) was added to reach a concentration of 125 mM. The pH value of the solution was subsequently adjusted to 1.58 using an aqueous solution of sodium hydroxide (Sigma-Aldrich, 97%). Sodium sulfate (Sigma-Aldrich, 99.0%) was added to the solution at a concentration of 70 mM. Subsequently, 35 ml of the resulting precursor solution was transferred into a 50 ml Teflon-lined stainless-steel autoclave and heated at 200°C for 24 h. The obtained nanowires were rinsed with deionized (DI) water and collected by centrifugation. To minimize potential contamination, this washing and centrifugation process was repeated 5 times with DI water and ethanol. The purified nanowires were dispersed in absolute ethanol, and a droplet of the resulting suspension was placed onto a TEM grid, followed by drying under ambient conditions.

### HAADF–STEM, STEM–EDS, 4D-STEM and STEM–EELS

The synthesized *h*-WO_3_ nanowires were characterized using a monochromated TEM (Titan Cubed G2, Thermo Fisher Scientific Inc., USA). ADF STEM imaging, STEM-energy dispersive X-ray spectroscopy (EDS), monochromated STEM–EELS and 4D-STEM were employed to assess samples’ morphology, elemental composition, *E*_g_ and local microstructure, respectively. For all measurements, the acceleration voltage for the incident electron beam was set to 300 kV. ADF–STEM images were obtained by detecting high-angle scattered electrons with a convergent illumination semi-angle of 17.9 mrad and an annular collection semi-angle of 52–200 mrad. STEM–EDS measurements were performed under the same incident electron beam conditions as ADF–STEM imaging. The 4D-STEM datasets were recorded using an electron probe with 0.6 mrad convergence semi-angle and a scanning step size of 2 nm. The electron energy loss (EEL) spectra were obtained using a GIF Quantum 965 spectrometer (Gatan Inc., USA) with an energy resolution of 0.15 eV, which was determined from the full width at half maximum of the zero-loss peak. To minimize electron-beam-induced damage to the *h*-WO_3_ nanowires during 4D-STEM and STEM–EELS measurements, all data were acquired with a probe current of 2.3 pA.

### SEM and SEM–CL

The SEM images of the *h*-WO_3_ nanowires were obtained using a field-emission SEM (Ultra, Carl Zeiss, Germany). The acceleration voltage and probe current were set to 2 kV and 0.5 nA, respectively. The CL spectra of the *h*-WO_3_ nanowires were acquired using an SEM (SU6600, HITACHI High-Tech., Japan) equipped with a spectrometer (MonoCL4, Gatan Inc., USA). In the MonoCL4 system, a retractable parabolic mirror and a light guide are used to collect the luminescence emitted from the sample. The acceleration voltage and probe current were set to 5 kV and 1 nA, respectively.

### DFT calculation

To investigate the electronic structure of *h*-WO_3,_ first-principles calculations based on density functional theory (DFT) were performed using the Vienna *Ab initio* Simulation Package [36–38], which employs a plane-wave basis set. The projector augmented wave (PAW) method was used to describe the electron–ion interactions. The Perdew–Burke–Ernzerhof formulation within the generalized gradient approximation was adopted for the exchange and correlation functional [39]. A plane-wave energy cutoff of 500 eV was applied. PAW potentials were used for all atoms, considering 6 valence electrons for oxygen (2s^2^2p^4^) and 12 valence electrons for tungsten (5p^6^5d^4^6s^2^).

The structural optimizations were carried out through the following procedure. First, the lattice spacing of the (0001) planes (*d*_0001_) was fixed at 0.377, 0.382 and 0.400 nm, corresponding to the values observed by 4D-STEM. Next, the (01$\overset{-}{1}$0) lattice spacing (i.e. the *a *=* b* lattice parameters) was systematically varied in increments of 0.02 nm. At each step, the positions of W and O atoms were relaxed to determine the most energetically favorable configuration. This process was repeated for all fixed *d*_0001_ values, and the structure with the lowest total energy was adopted for subsequent electronic structure ­analysis. The convergence criterion for structural relaxation was set to ensure that the force acting on each ion was less than 0.01 eV·Å^−1^.

For electronic structure calculations, the total energy convergence criterion was set to less than 1.0 × 10^−6 ^eV. The *k*-point mesh for Brillouin zone sampling was generated using a spacing of 2π  ×  0.02 Å^−1^, centered at the *Γ*-point in all calculations. The DFT+*U* approach was employed to address the underestimation of the bandgap in WO_3_ due to self-interaction errors inherent in standard DFT. This approach introduces an on-site Coulomb correction (*U*) to more accurately describe the strong electron–electron interactions in localized *d* orbitals. In this study, following the approach of Zhang *et al*. [40], a value of *U *= 6.2 eV was applied to the W 5d orbitals.

To elucidate the nature of electronic bonding interactions in *h*-WO_3_, we performed a COHP analysis [41] using the local orbital basis suite toward electronic structure reconstruction code [42–44].

Noted that standard DFT calculations provide access to ground-state electronic structures, whereas experimental techniques such as EELS and CL probe excited-state transitions. Therefore, the comparison between DFT calculations and EELS and CL measurements presented in this study is intended to capture qualitative trends in electronic structure, rather than to reproduce absolute excitation energies.

## Results and discussion

### Morphological and compositional characterization

**Fig. 1.**
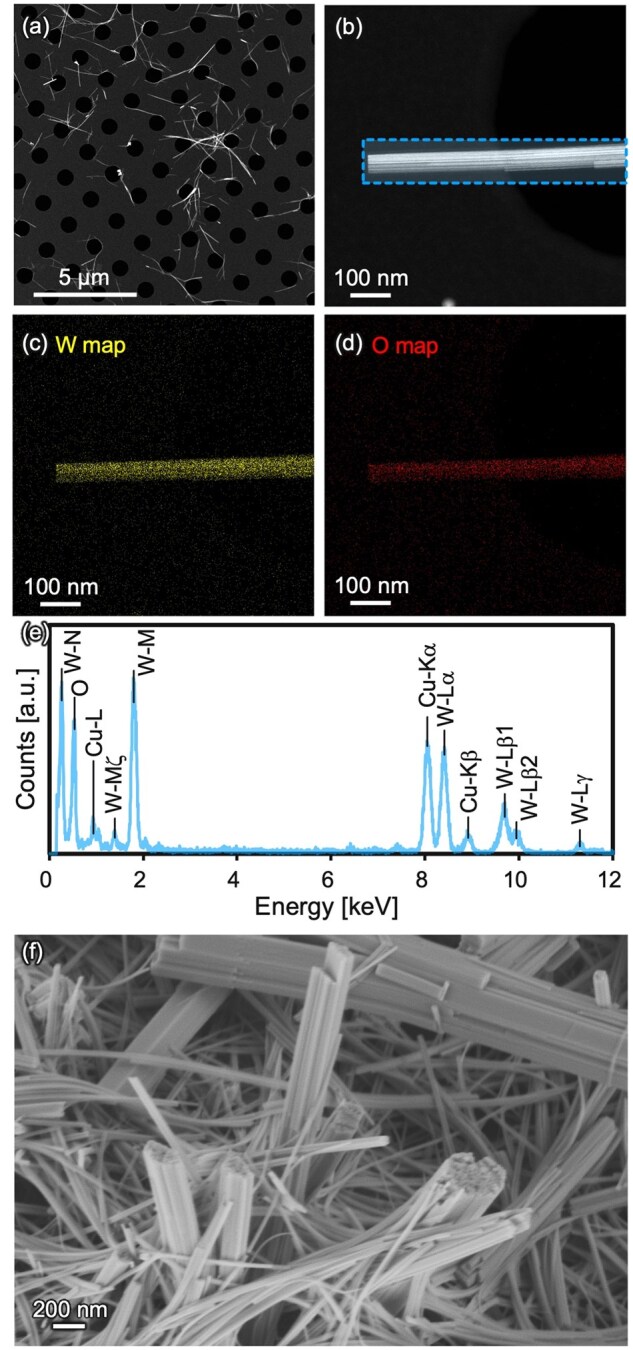
ADF–STEM images of (a) *h*-WO_3_ nanowires and (b) a single *h*-WO_3_ nanowire, STEM–EDS elemental maps for W (c) and O (d) in the same area as (b), and (e) an EDS profile acquired from a rectangular area denoted by a broken line in (b). (f) SEM image of *h*-WO_3_ nanowires.

Figure 1 presents the ADF–STEM images, EDS elemental maps and SEM image of the obtained sample. The ADF–STEM image demonstrated numerous nanowires dispersed on the TEM microgrid. Each nanowire has a diameter of several tens of nanometers and a length of several micrometers. Some nanowires exhibited noticeable bending. The STEM–EDS elemental maps and EDS profiles confirmed that the nanowires consist exclusively of tungsten and oxygen. A copper (Cu) signal originating from the TEM microgrid substrate was also detected. The SEM image of the nanowires revealed that their side surfaces are irregular rather than smooth, exhibiting a morphology resembling bundle of fine strands. This characteristic surface texture may contribute to the band-like contrasts observed in the ADF–STEM images, reflecting subtle variations in surface structure or thickness.

### Analysis of the crystal structure of *h*-WO_3_ nanowires

**Fig. 2.**
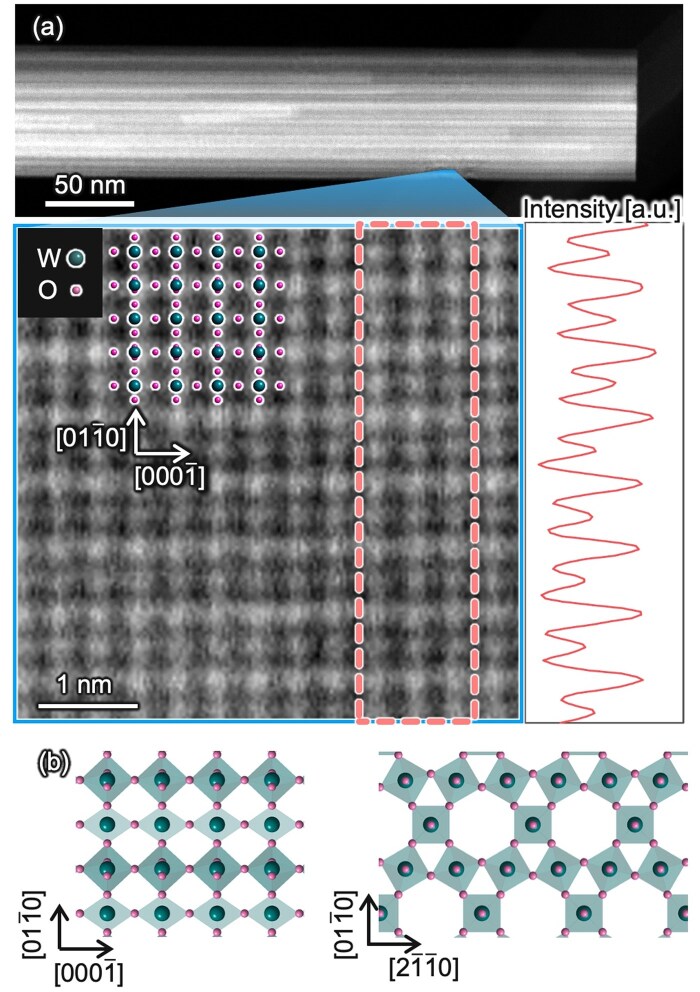
(a) ADF–STEM images of a single *h*-WO_3_ nanowire viewed along [2$\overset{-}{1}\overset{-}{1}$0] and an intensity profile of the W atomic columns along [01$\overset{-}{1}$0], (b) crystal structures of *h*-WO_3_ viewed along [2$\overset{-}{1}\overset{-}{1}$0] (left) and [000$\overset{-}{1}$] (right).

The synthesized nanowires exhibited columnar contrast along [0001] (Fig. 2a), which could stem from 3 possible origins. First, the contrast may result from the aggregation of multiple single-crystalline nanowires into a bundled configuration. In this case, the adjacent rod-like domains would be slightly rotated around the [0001] axis, forming low-angle grain boundaries. These boundaries can locally enhance or suppress high-angle scattering in the ADF–STEM image. Second, if the nanowire is single crystalline, similar band-like contrasts could emerge from variations in surface inclination or faceting. These variations in surface morphology alter the local channeling conditions of the incident electron beam, resulting in contrast variations in the image. In addition, the presence of inherent “wheel-like” crystallographic defects in *h*-WO_3_, as previously reported by Besnardière *et al*. [45], may also contribute to the formation of such features. The “wheel-like” structure consists of 6 WO_6_ octahedra connected at their corners, forming a hexagonal ring when viewed along [0001]. These units are periodically stacked along [0001] to create 1-dimensional tunnels with an internal diameter of approximately 0.48 nm. Additional 4- and 3-membered tunnels, which are absent in conventional *h*-WO_3_, are also present. These defects often form during low-temperature *h*-WO_3_ synthesis.

A closer examination of the high-resolution ADF–STEM image revealed an alternating image-intensity modulation of tungsten atomic columns along [01$\overset{-}{1}$0]. This contrast modulation is attributed to the tunnel-like structure formed by the periodic arrangement of WO_6_ octahedra within the {0001} planes (Fig. 2b). This structure is consistent with the characteristics of *h*-WO_3_ in the space group *P*6/*mmm* [45, 46].

### 4D-STEM analysis of lattice distortion in bent *h*-WO_3_ nanowires

**Fig. 3.**
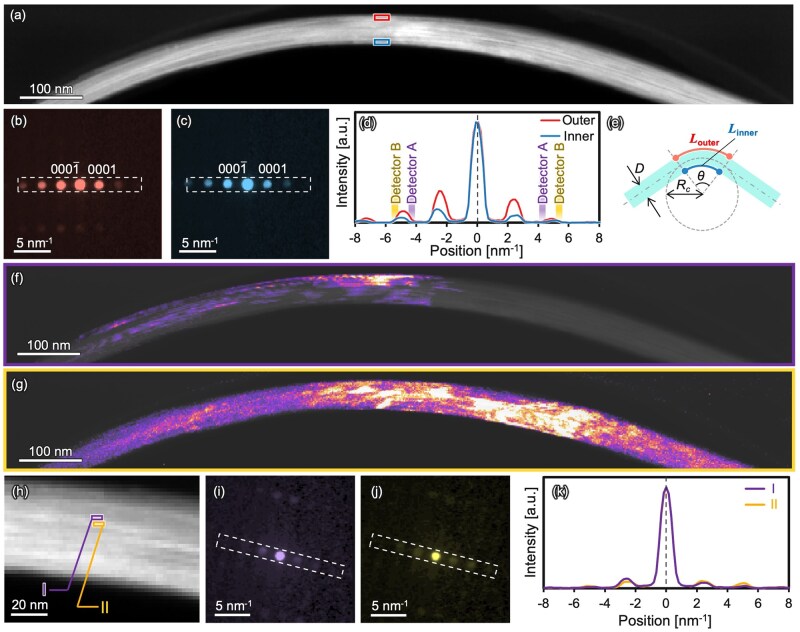
4D-STEM dataset of a single *h*-WO_3_ nanowire with a 44-nm diameter and a 1.2-μm radius of curvature. (a) ADF–STEM image. (b, c) Electron diffraction patterns of the outer and inner rectangular areas respectively in (a). (d) Line profiles of the diffraction spots in (b) and (c). (e) Geometric model for the relationship between the curvature and 2 arc lengths of the outer (*L*_outer_) and inner (*L*_inner_) in a bending position of the wire structure. (f, g) Virtual DF–STEM images from virtual annular DF detectors named as detectors A and B in (d). These collection semi-angles are 8.1–8.9 mrad (4.1–4.5 nm^−1^, corresponding to 0.22–0.24 nm in real space) and 10.2–11.0 mrad (5.2–5.6 nm^−1^, corresponding to 0.18–0.19 nm in real space), respectively. (h–k) Local structural analysis of the band-like contrast region shown in (a). (h) Enlarged ADF–STEM image highlighting the selected regions (I and II). (i, j) Electron diffraction patterns extracted from regions I and II, respectively. (k) Line profiles of the diffraction spots in (i) and (j). A slight difference in lattice spacing was observed between the 2 regions, indicating that local lattice relaxation occurred near the band-like contrast.

To investigate the structural deformation in bent *h*-WO_3_ nanowires, a 4D-STEM dataset was acquired from a 44-nm-­diameter nanowire and a curvature radius of 1.2 μm (Fig. 3a). Figure 3b and c presents superimposed electron diffraction patterns collected from the rectangular regions (4 × 10 nm) on the outer and inner sides of the curved segment, as denoted, respectively, in Fig. 3a. By averaging diffraction patterns from multiple points within each region, random noise effects were reduced, enhancing diffraction feature clarity. Comparing Fig. 3b and Fig. 3c, the diffraction pattern differences between the outer and inner bent regions of the nanowire are evident, implying that the nanowire undergoes not only simple flexural deformation but also a degree of torsional strain during bending. Figure 3d shows the positions of the diffraction intensity maxima determined from the [0001] zone axis diffraction intensity profiles in Fig. 3b and c. To ensure accurate peak position determination, each diffraction spot was extracted by applying 2-dimensional Gaussian fitting. The distances from the origin of the reciprocal lattice to the *hkil *= 0001 diffraction spots were 2.50 and 2.65 nm^−1^ for the outer and inner bend sides, respectively. These diffraction spots correspond to (0001) lattice spacings (*d*_0001_) of 0.400 and 0.377 nm, respectively. In contrast, a straight nanowire segment exhibited a *d*_0001_ of 0.382 nm (Supplementary Data A). These results indicate that the *h*-WO_3_ nanowire curvature primarily results from lattice expansion on the outer side of the bent segment.

A geometric model of bending deformation is illustrated in Fig. 3e. For a nanowire of diameter *D* bent with a curvature radius *R*_c_, the arc lengths of the outer and inner surfaces, *L*_outer_ and *L*_inner_,are given by $(R_{c}+\;\frac{1\;}{2}D)\;\theta \;$ and $(R_{c}-\;\frac{1\;}{2})\;\theta$ respectively, where θ is the central angle of the curvature (Fig. 3e). Thus, the ratio of outer to inner arc lengths is calculated as follows:


(1) LouterLinner=2Rc + D2Rc - D .

Assuming no crystallographic defects such as dislocations or vacancies, the geometric strain can be interpreted as an expansion or contraction of the interplanar spacing along the long axial direction.

For the bent *h*-WO_3_ nanowire in Fig. 3a, with diameter *D *= 44 nm and curvature radius *R*_c_ = 1.2 μm, the geometric model yields a predicted lattice strain of approximately 3.7%, based on the arc length ratio. However, the experimental measurements of *d*_0001_ at the outermost and innermost regions of the nanowire were 0.400 and 0.377 nm, respectively. These values correspond to an actual lattice strain of 6.10%, which exceeds the value predicted by the geometrical model (3.7%).

Figure 3f and g displays virtual ADF–STEM images from the 4D-STEM dataset using 2 virtual detectors: A (scattering angle 8.1–8.9 mrad, corresponding to 0.22–0.24 nm in real space) and B (10.2–11.0 mrad, corresponding to 0.18–0.19 nm in real space). Note that all diffraction patterns were subjected to binning, causing the diffraction spots to extend across several pixels. Consequently, the detectors may capture the intensity from the surrounding tails. As shown in Fig. 3f, a region where the diffraction spot overlaps with the detector range corresponding to a *d*_0002_ spacing of 0.22–0.24 nm is localized on the outer side of the top central part of the bent segment. This spatially confined expansion of the lattice spacing suggests that lattice distortions are not uniformly distributed throughout the bent region. Such localized lattice expansions likely contribute significantly to the emergence of the unexpectedly large strain (6.1%) observed in the experiment. In contrast, regions with a narrower *d*_0002_ spacing of 0.18–0.19 nm were distributed throughout the nanowire (Fig. 3g).

Although the geometric model predicts a uniaxial strain of approximately 3.7% at the outermost region of the bent nanowire, the experimental measurements revealed a larger strain of 6.10%. This difference implies that local deformation in the bent region is more complex than that predicted by a purely elastic bending model. As shown in Fig. 3h, an enlarged ADF–STEM image highlights a representative band-like contrast along the nanowire. Two regions labeled I (outer side) and II (inner side) were selected across this feature. Figure 3i and j shows diffraction patterns reconstructed from the 4D-STEM dataset, clearly resolving the 000*l* reflections. The corresponding line profiles (Fig. 3k) reveal that *d*_0001_ in region I is slightly smaller than that in region II, indicating localized structural relaxation on the tensile side. However, not all band-like contrasts exhibited this type of relaxation, suggesting that defect-induced strain redistribution does not occur uniformly throughout the nanowire. An example without a detectable change in *d*_0001_ is shown in Supplementary Data B. These results link the band-like contrasts to local strain perturbations and help to explain the discrepancy between the geometric model and experiment. In addition, torsional deformation inferred from diffraction patterns and local plastic deformation may influence the overall strain state of the nanowire [47, 48]. Further atomistic simulations and high-resolution structural characterization (e.g. by geometric phase analysis or *in situ* TEM) are needed to fully elucidate the interplay between these factors and their role in strain amplification.

Although these atomistic irregularities complicate the strain state in real systems, theoretical modeling based on ideal elastic deformation is essential for clarifying the fundamental impact of lattice strain on the electronic structure. Accordingly, the modulation of the electronic structure of *h*-WO_3_ nanowires in bent regions was investigated under the assumption of ideal elastic deformation.

### DFT calculations of bandgap variations induced by lattice strain

Changes in semiconductor volume and lattice constants can affect their electronic structure, including *E*_g_ [20–26]. The observed variation in *d*_0001_ in bent *h*-WO_3_ nanowires suggests a potential impact on *E*_g_. In II–VI and III–V compound semiconductors such as GaAs and ZnS, increased interplanar spacing often decreases *E*_g_ due to weaker interatomic bonding from volumetric expansion [20, 22, 25]. In contrast, in perovskites, where both the valence band maximum (VBM) and conduction band minimum (CBM) consist of antibonding orbitals, increased lattice constants can decrease orbital overlap, lowering VBM and CBM energy levels and potentially increasing *E*_g_. These examples show that the relationship between lattice spacing and *E*_g_ cannot be universally generalized [21, 26]. Furthermore, quantum confinement and surface effects in nanoscale semiconductors add further complexity to the *E*_g_ determination [23, 24].

To elucidate the influence of lattice distortion on the electronic structure of *h*-WO_3_, we performed DFT calculations and COHP analysis, focusing on *E*_g_ changes associated with *d*_0001_ variations. Although this model offers valuable insight into the relationship between uniaxial lattice expansion and bandgap narrowing, the actual bent nanowire likely experiences more complex anisotropic strain. Therefore, this model is an approximation that does not completely capture the strain complexity of the experimental system.

**Fig. 4.**
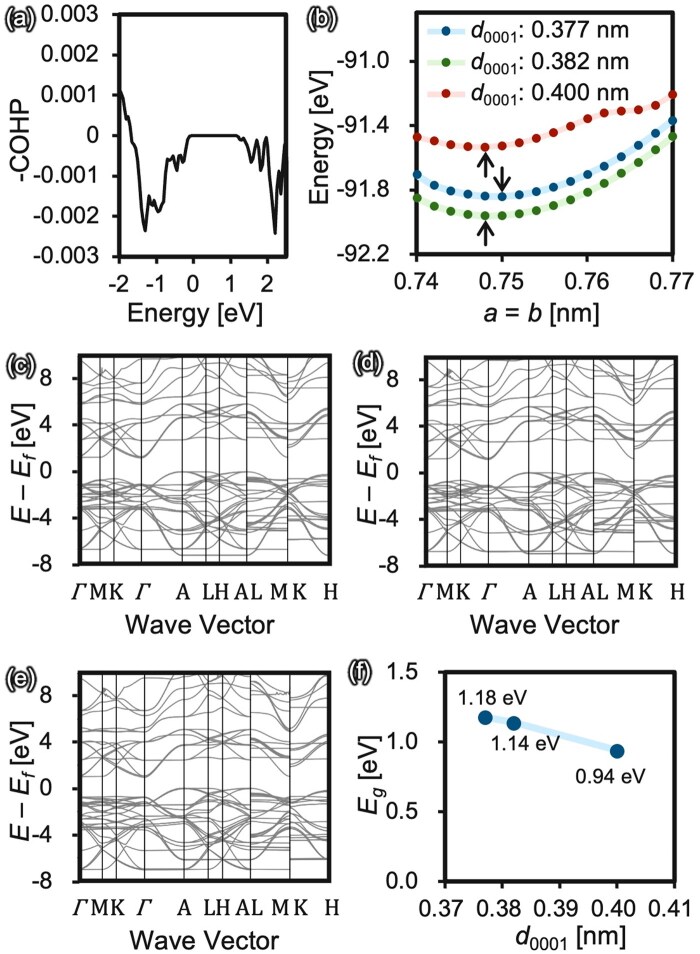
(a) –COHP diagram of all W–O bonds of *h*-WO_3_. Both the VBM and CBM originate from antibonding orbitals. (b) Total energy of *h*-WO_3_ with varying lattice parameter *a *=* b* at fixed values of *d*_0001_. The arrows indicate the most stable *a *=* b* values for each *d*_0001_. (c–e) Calculated *h*-WO_3_ energy band structures. The value of *d*_0001_ was set (c) 0.377, (d) 0.382, and (e) 0.400 nm. (f) Bandgap energy dependence on the value of *d*_0001_, showing a systematic decrease in *E*_g_ with increasing *d*_0001_.

Figure 4a shows the COHP analysis results for all W–O covalent bonds in *h*-WO_3_, with the reference zero point being the VBM energy. The –COHP diagram revealed that both VBM and CBM have negative values, indicating antibonding orbitals. Thus, unlike in typical II–VI or III–V semiconductors, the elongation of interplanar spacing in *h*-WO_3_ does not necessarily decrease *E*_g_. Subsequently, we constructed energetically optimized crystal structures of *h*-WO_3_. Figure 4b shows the calculated minimum total energies as a function of the *a *=* b* values. The most stable *a *=* b* value was found to be 0.748 nm for *d*_0001_ = 0.382 and 0.400 nm, and 0.750 nm for *d*_0001_ = 0.377 nm. The calculated electronic band structures are presented in Fig. 4c–e. In all cases, *h*-WO_3_ exhibited an indirect bandgap located between the *A* and *Γ* points of the Brillouin zone, regardless of the *d*_0001_ value. Notably, the calculated *E*_g_ systematically decreases with increasing *d*_0001_ (Fig. 4f). These results indicate that uniaxial expansion along the [0001] direction is associated with a systematic reduction in the bandgap of *h*-WO_3_. This trend suggests that *E*_g_ is narrowed in the outer regions of bent *h*-WO_3_ nanowires.

### Experimental verification of bandgap energy by STEM–EELS and CL analysis

**Fig. 5.**
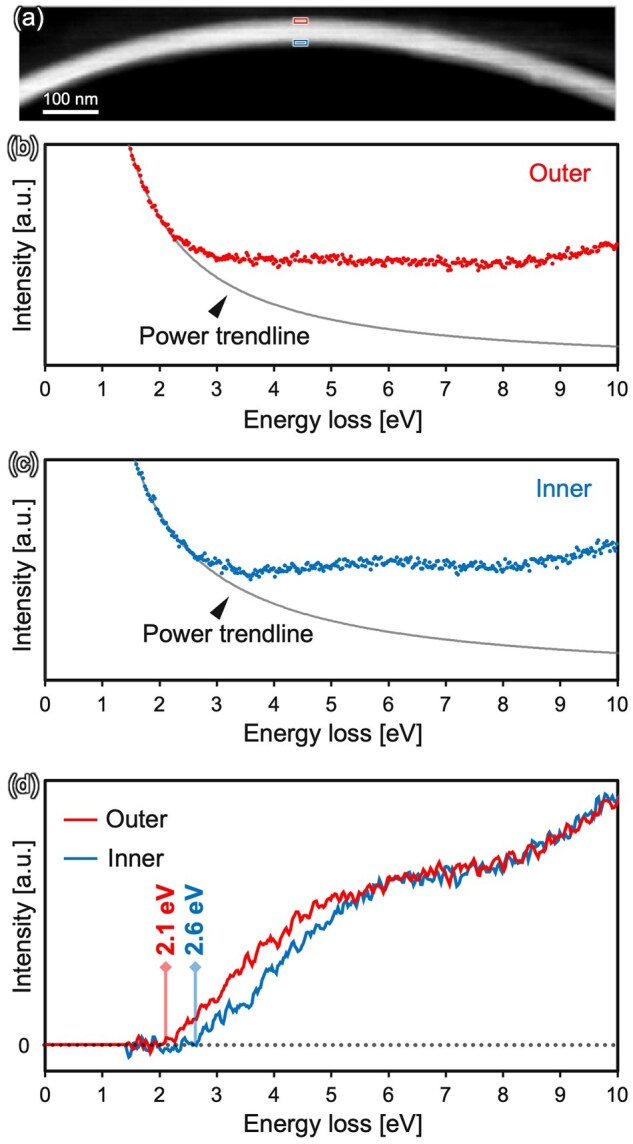
STEM–EELS dataset of a single *h*-WO_3_ nanowire with a 44-nm diameter and a 1.2-μm radius of curvature. (a) STEM–ADF image. (b, c) EEL spectra (dots) obtained from the outer and inner rectangular areas indicated in (a), together with the fitted power-law trendlines in the energy range of 0.5–1.5 eV (gray curves). (d) ZLP-subtracted EEL spectra of the outer and inner rectangular areas in (a).

To experimentally validate the DFT-predicted relationship between interplanar spacing and electronic structure, we performed spatially resolved measurements of the bandgap energy using monochromated STEM–EELS. In semiconductors, the lowest onset of energy loss in an EEL spectrum typically corresponds to the optical *E*_g_ [12]. A monochromated electron beam was employed to reduce the influence of the ZLP tail on the measurement of *E*_g_. Figure 5b and c shows the EEL spectra acquired from the same region of the *h-*WO_3_ nanowire analyzed in the 4D-STEM dataset shown in Fig. 3. The EEL spectra were extracted within rectangular regions measuring 4 × 10 nm on the outer and inner sides of the bend (Fig. 5a). The spectra shown in Fig. 5d were obtained after subtracting the zero-loss peak (ZLP) component. The ZLP tail was fitted using a power-law function in the energy range of 0.5–1.5 eV to isolate the bandgap onset. The power-law fitting is widely used in EELS studies [17, 49]. It reproduces the gradual decay of the ZLP tail caused by instrumental broadening and quasi-elastic scattering, and it helps to avoid over-subtraction of the signal near the bandgap region.

As shown in Fig. 5d, the spectral onset at the outer and inner sides of the bend was measured to be 2.1 and 2.6 eV, respectively. These values approximate the local *h*-WO_3_ bandgap energies in the respective regions. The onset at the inner side (2.6 eV) aligns well with the previously reported optical bandgap of *h*-WO_3_ (2.6–2.7 eV) [50, 51]. However, the onset determination may be influenced by inelastic scattering processes such as Cherenkov radiation [52] and surface plasmon excitations [53], which cannot be entirely excluded under these experimental conditions. Therefore, the extracted onset values should be considered qualitative indicators of *E*_g_ rather than definitive quantitative measurements. Importantly, the observed reduction in onset energy at the outer side of the bend is consistent with DFT predictions that tensile strain along the [0001] direction (i.e. increased *d*_0001_ spacing) leads to bandgap narrowing. This trend was reproducibly observed in several other bent *h*-WO_3_ nanowires, as presented in Supplementary Data C, indicating that the reduction of the onset energy at the outer side is a general feature associated with bending-induced tensile strain.

**Fig. 6.**
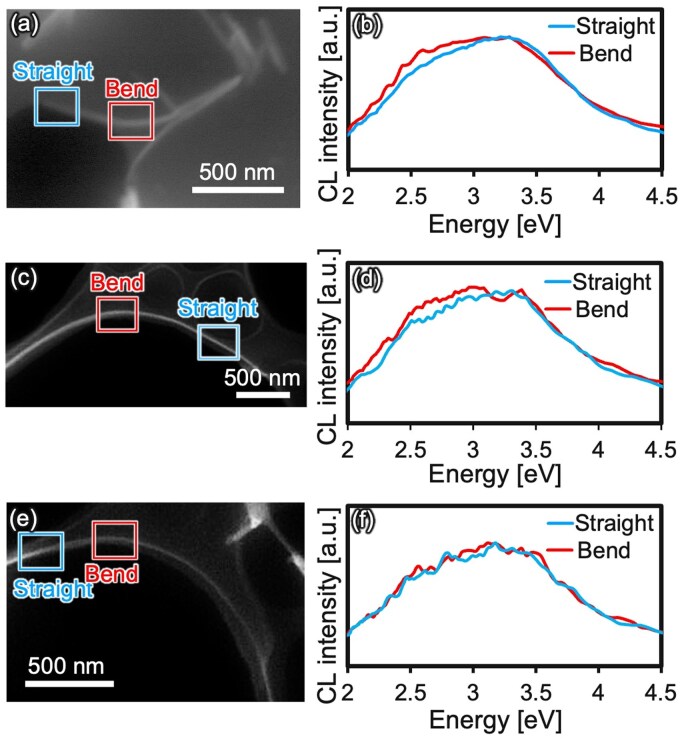
SEM–CL analysis of *h*-WO_3_ nanowires. (a, c, e) SEM images showing the straight and bent regions. (b, d, f) Corresponding CL spectra obtained from the regions indicated in (a), (c), and (e), respectively.

CL measurements performed on both straight and bent regions of the *h*-WO_3_ nanowire further validated the results of the EELS measurements. As shown in Fig. 6b and d, clear redshifts of the CL spectra were observed in the bent region. These redshifts indicate that *E*_g_ is reduced in the bent region. In contrast to STEM–EELS, SEM–CL involves a much larger interaction volume and typically offers spatial resolution on the order of several tens to hundreds of nanometers [11]. Although CL cannot resolve highly localized bandgap variations, it is well-suited for capturing spatially averaged spectral trends across larger regions. This complementary measurement thus reinforces the finding from the EELS measurement that lattice expansion leads to bandgap narrowing in *h*-WO_3_ nanowires. However, a redshift was not consistently observed in all bent *h*-WO_3_ nanowires. As shown in Fig. 6f, some exhibited almost no spectral change. This variability may arise from differences in the degree of local lattice expansion in the bending *h*-WO_3_ nanowires. In addition, because CL represents a spatially averaged response, localized electronic variations may not always be clearly resolved. As a result, some nanowires exhibited weaker or even undetectable spectral shifts.

### Comparison between experimental and theoretical bandgap variations

EELS and CL measurements evaluate the optical *E*_g_, which includes excitonic effects. In contrast, DFT calculations provide the single-particle *E*_g_ for the ground state. Consequently, a direct comparison of the absolute *E*_g_ values obtained from these 2 approaches is inherently limited. Furthermore, it is well established that standard DFT calculations systematically underestimate the value of *E*_g_ compared to experimental measurements. This underestimation arises because standard DFT is a ground-state theory and the commonly used exchange–correlation functionals fail to account for the discontinuity in the exchange–correlation potential and many-body effects. Consistent with this established tendency, the calculated *E*_g_ in the present study was found to be approximately half the experimentally measured value. Instead of directly comparing the absolute values, the ratio of the *E*_g_ on the outer side to the inner side is calculated to provide a more meaningful point of comparison between theory and experiment. Our DFT calculations show the *E*_g_ of 1.18 eV on the inner side and 0.94 eV on the outer side, giving an outer-to-inner ratio of ∼0.80. On the other hand, the experimental EELS data show the *E*_g_ of 2.6 eV on the inner side and 2.1 eV on the outer side, yielding a remarkably similar ratio of ∼0.81. The relative agreement between our calculations and experiments confirms that this DFT approach successfully reproduces the fundamental physics of the band structure changes under strain in the bending *h*-WO_3_ nanowires.

The exciton binding energy (*E*_b_) in WO_3_ has been reported to be approximately 8–18 meV [54]. This parameter is expected to exhibit some degree of variation when strain is introduced. However, no evidence has been reported to date of *E*_b_ changing by a magnitude comparable to the experimentally observed spectral shifts of hundreds of meV within this material system. Therefore, the significant spectral shifts observed in EELS and CL are unlikely to be predominantly caused by variations in *E*_b_. Nevertheless, a more rigorous treatment of optically excited states is still required to fully account for exciton binding effects. This step is essential for the persistent gap between theoretical estimations and experimental observations, and remains a task for future work.

### Implications of strain-induced bandgap modulation

Since *E*_g_ variations influence carrier transport and ion storage behavior [55], controlled deformation offers a viable method for tuning the functionality of flexible electronic and energy devices. The observed mechanism of bandgap modulation in bent *h*-WO_3_ nanowires—which involves strain-induced changes in atomic arrangements, lattice constants and the electronic structure—is likely applicable to other MO_*x*_ semiconductors and low-dimensional materials with similar bonding characteristics. These findings highlight the broad potential of strain engineering for tuning the properties of materials across diverse systems. They also suggest promising pathways for developing innovative devices, such as strain-tunable optoelectronic devices and spatially programmable ion storage platforms.

## Concluding remarks

In this study, we investigated the correlation between local structural variations and electronic structure in bent regions of *h*-WO_3_ nanowires using a combination of several different electron microscopy experiments and first-principles DFT calculations.

4D-STEM provided high-spatial-resolution insight into lattice distortions, revealing asymmetric expansion of the interplanar spacing (*d*_0001_) in the outer region of the bent segments. While a simple geometric model qualitatively captured the trend of lattice variations, the actual distortions we observed experimentally were significantly larger than the model’s predictions. This discrepancy highlights the limitations of a simple geometric model and underscores the necessity of direct experimental validation at the nanoscale.

Monochromated STEM–EELS measurements confirmed the electronic consequences of these distortions, revealing a clear reduction in *E*_g_ on the outer side of the bend (tensile region). This finding was further corroborated by CL spectroscopy, which exhibited a redshift in the emission spectrum. Furthermore, DFT calculations qualitatively reproduced the systematic narrowing of the bandgap with increasing *d*_0001_ spacing. This provides a theoretical basis for how strain modulates the electronic structure.

This study highlights the potential of local strain as a design parameter for future nanoelectronic and optoelectronic applications. Furthermore, these methodologies can broadly be applied to other low-dimensional materials to investigate strain–property relationships at the nanoscale.

## Supplementary Material

dfaf050_Supplementary_Data
