# Supplementary material for: Local strain effects on bandgap energy in flexible h-WO3 nanowires
Source: Microscopy (Oxf). 2025 Oct 31;75(4):332–42. doi: 10.1093/jmicro/dfaf050 (PMC13454687; doi:10.1093/jmicro/dfaf050)
Supplement: Supplementary file 1 [file dfaf050_supplementary_data.docx]

**Supplementary Data**

**Local Strain Effects on Bandgap Energy in Flexible *h*-WO_3_ Nanowires**

Sho Nekita^1*^, Naomu Sekiguchi^1^, Yuya Kasamura^1,2^, Itsuki Misono^1^, Yusuke Shimada^3^, Satoshi Iikubo^3^, Tetsuya Okuyama^3^, Satoshi Hata^3,4^

^1^Interdisciplinary Graduate School of Engineering Sciences, Kyushu University, 6-1 Kasugakoen, Kasuga, Fukuoka 816-8580, Japan

^2^Panasonic Industry Co., Ltd., 1006, Oaza Kadoma, Kadoma, Osaka 571-8506, Japan

^3^Faculty of Engineering Sciences, Kyushu University, 6-1 Kasugakoen, Kasuga, Fukuoka 816-8580, Japan

^4^The Ultramicroscopy Research Center, Kyushu University, 744 Motooka, Nishi, Fukuoka 819-0395, Japan

*Corresponding author

Phone: +81-092-583-7580

E-mail: nekita.sho.694@s.kyushu-u.ac.jp

**Supplementary Data:**

**A. (0001) Lattice Spacing in the Straight Region of the *h*-WO_3_ Nanowire**

**B. Example of a Band-Like Contrast Region Without Detectable *d*_0001_ Variation**

**C. Reproducibility of the EELS Onset Energy Reduction in Bent *h*-WO_3_ Nanowires**

**A. (0001) Lattice Spacing in the Straight Region of the *h*-WO_3_ Nanowire**

The lattice spacing of the (0001) in the straight segment of the *h*-WO_3_ nanowire was evaluated using 4S-STEM. The measurement conditions were identical to those used for the curved region, as shown in Fig. 3. The distances from the origin of the reciprocal lattice to the *hkil* = 0001 diffraction spots was measured to be 2.62 nm⁻¹. This diffraction spots correspond to (0001) lattice spacings of 0.382 nm.

**Figure S1.** 4D-STEM dataset of a single *h*-WO_3_ nanowire with a 44-nm diameter. (a) ADF-STEM image, (b) electron diffraction pattern of rectangular areas in (a). (c) A line profile of the diffraction spots in (b).

**B. Example of a Band-Like Contrast Region Without Detectable *d*_0001_ Variation**

As shown in Figure S2(a), an enlarged ADF–STEM image highlights the analyzed region across the band-like contrast. Figure S2(b) and S2(c) show 4D-STEM diffraction patterns integrated respectively in the two adjacent regions showing different ADF-STEM image intensities denoted in Figure S2(a). The corresponding intensity profiles along the 000*l* systematic row in Figure S2(d) show nearly identical peak positions, indicating that the lattice spacing *d*_0001_ remained unchanged within the experimental precision. This observation confirms that not all band-like contrast originate from lattice defects, but rather reflect variations in surface morphology.

**Figure S2.** Local structural analysis to discuss the origin of the band-like contrast. (a) ADF–STEM image highlighting the selected regions (I and II). (b, c) Electron diffraction patterns extracted from regions I and II, respectively. (d) Line profiles of the diffraction spots in (b) and (c). No difference in lattice spacing was detected. This indicates no local lattice relaxation near the band-like contrast.

**C. Reproducibility of the EELS Onset Energy Reduction in Bent *h*-WO_3_ Nanowires**

The onset energy of the EEL spectrum was evaluated for additional bent *h*-WO_3_ nanowires to confirm the reproducibility of the results shown in Fig. 5. STEM–EELS measurements were performed under the same acquisition conditions as those described in the main text. For each nanowire, the EEL spectra obtained from the outer and inner sides of the bent region are shown in Figs. S3(b–d) and S4(b–d). The spectral onset at the outer side was consistently lower than that at the inner side, indicating a reproducible reduction in the local bandgap energy.

**Figure S3.** STEM–EELS dataset for a single *h*-WO_3_ nanowire with a 31-nm diameter and a 0.8-μm radius of curvature. (a) STEM–ADF image. (b, c) EEL spectra (dots) obtained from the outer and inner rectangular areas indicated in (a), together with the fitted power-law trendlines in the energy range of 0.5–1.5 eV (gray curves). (d) ZLP-subtracted EEL spectra of the outer and inner rectangular areas in (a).

**Figure S4.** STEM–EELS dataset for a single *h*-WO_3_ nanowire with a 19-nm diameter and a 1.4-μm radius of curvature. (a) STEM–ADF image. (b, c) EEL spectra (dots) obtained from the outer and inner rectangular areas indicated in (a), together with the fitted power-law trendlines in the energy range of 0.5–1.5 eV (gray curves). (d) ZLP-subtracted EEL spectra of the outer and inner rectangular areas in (a).
